# Integrating cellular senescence with the concept of damage accumulation in aging: Relevance for clearance of senescent cells

**DOI:** 10.1111/acel.12841

**Published:** 2018-10-22

**Authors:** Mikolaj Ogrodnik, Hanna Salmonowicz, Vadim N. Gladyshev

**Affiliations:** ^1^ Institute for Cell and Molecular Biosciences Newcastle University Institute for Ageing Newcastle upon Tyne UK; ^2^ Division of Genetics Department of Medicine Brigham and Women's Hospital and Harvard Medical School Boston Massachusetts

**Keywords:** aging, cellular senescence, deleteriome, evolutionary biology, lifespan, molecular damage, theories of aging

## Abstract

Understanding the aging process and ways to manipulate it is of major importance for biology and medicine. Among the many aging theories advanced over the years, the concept most consistent with experimental evidence posits the buildup of numerous forms of molecular damage as a foundation of the aging process. Here, we discuss that this concept integrates well with recent findings on cellular senescence, offering a novel view on the role of senescence in aging and age‐related disease. Cellular senescence has a well‐established role in cellular aging, but its impact on the rate of organismal aging is less defined. One of the most prominent features of cellular senescence is its association with macromolecular damage. The relationship between cell senescence and damage concerns both damage as a molecular signal of senescence induction and accelerated accumulation of damage in senescent cells. We describe the origin, regulatory mechanisms, and relevance of various damage forms in senescent cells. This view on senescent cells as carriers and inducers of damage puts new light on senescence, considering it as a significant contributor to the rise in organismal damage. Applying these ideas, we critically examine current evidence for a role of cellular senescence in aging and age‐related diseases. We also discuss the differential impact of longevity interventions on senescence burden and other types of age‐related damage. Finally, we propose a model on the role of aging‐related damage accumulation and the rate of aging observed upon senescent cell clearance.

## 1 WHY “CURING” AGING IS SO DIFFICULT? MEET THE UNAVOIDABLE DAMAGE ACCUMULATION PROCESS

The aging process affects organisms from the smallest bacteria to the largest animals and is thought to be universal for organisms with the separation of germline and soma. However, some symmetrically dividing cells (e.g., bacteria, fungi, or cancer cells) appear to escape the consequences of aging. The aging process may also not be visible for animals characterized by the so‐called negligible senescence, such as a species within the genus of *Cnidaria*—Hydra, although it is known that their individual cells do age (Danko, Kozlowski & Schaible, 2015; Stewart, Madden, Paul & Taddei, 2005). This apparent nonaging phenotype can be achieved by replacing cells that accumulated damage over time with new cells generated from abundant stem cells that can give rise to any cell type in the body. However, this nonaging strategy is not applicable to the great majority of organisms with specialized, nonreplaceable cells and structures. When organisms are unable to replace cells at will or dilute damage, intracellular damage accumulates, exerting its deleterious effect on the host cell as well as other cells, impairing their function and ultimately contributing to age‐related diseases and to aging itself. The macroscopic age‐associated changes in organisms are so obvious and severe that identifying their molecular bases would seem to be an easy task. Yet, all the research conducted so far has not led to the unambiguous identification of the causal factors orchestrating aging.

Why are the processes responsible for aging so difficult to pinpoint? Since the early days of research on aging, it has been repeatedly observed that old organisms accumulate modified macromolecules and various side products of housekeeping processes. Such age‐related macromolecular modifications are considered to be manifestations of “imperfectness” of molecular systems; thereby, any biologic function bears the risk of producing faulty products (Gladyshev, 2013). Even without any interaction, macromolecules can acquire structural modifications due to external and internal stress stimuli (Kirkwood, 2005), spontaneous chemical reactions, or simply due to stochastic processes, ultimately leading to an increase in entropy of the system (Truscott, Schey & Friedrich, 2016; Zimniak, 2008). These structural modifications and their increase over time can be called “damage” and “damage accumulation,” respectively. It should be noted, however, that in the scientific literature, the term “damage” is often used quite superficially and usually does not encompass the whole variety of modifications that molecules acquire with age and the consequent deleterious changes they accumulate. It is the sum of all deleterious changes, including molecular damage, that represents the aging process.

**Figure 1:**
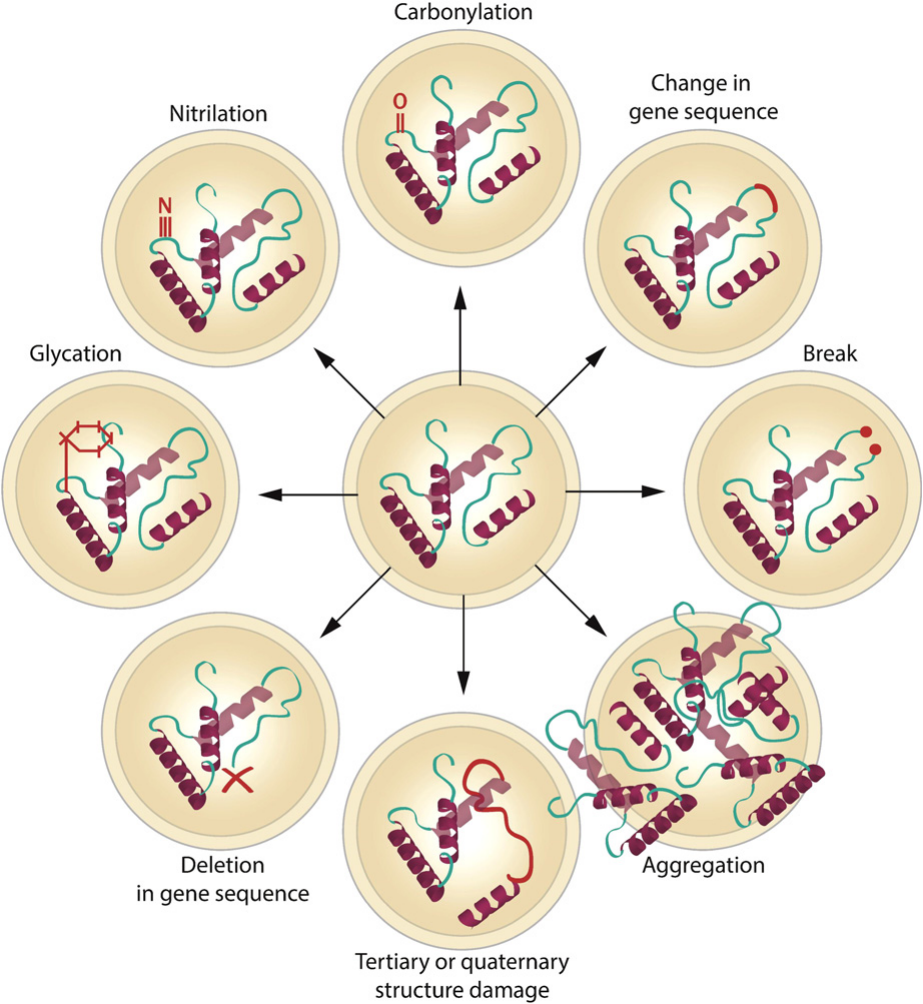
**Examples of macromolecular damage.** A single macromolecule (protein) can be affected by a wide variety of damage types whose heterogeneity is only partially understood. Different damage types, some of which are individually illustrated in the figure, can coexist in the same macromolecule and affect its functionality

Some of the most common types of damage that can affect protein function include oxidation, deamination, racemization, isomerization, carbonylation, and nitrilation, and all kinds of molecular breaks, adductions, substitutions, insertions, and deletions (Figure 1). Not only proteins but likely all biologic macromolecules are vulnerable to multiple types of modifications, although their chemical composition and structure make them more vulnerable to some insults more than others. Even a single class of molecules can display a great diversity of damage types it may acquire during aging. For instance, Hsp70 protein—a major molecular chaperone and folding catalyst—accumulates various forms of oxidative damage (Dukan & Nystrom, 1998; Tamarit, Cabiscol & Ros, 1998), while vimentin—type III intermediate filament protein—is known to accumulate glycation‐based modifications (Kueper et al., 2007). Considering heterogeneity of damage, two molecules of the same enzyme might lose function due to different structural modifications. For example, it has been observed that enzymes isolated from the liver lose their activity with age due to different patterns of damage accumulation: Malic enzyme loses on average a single histidine residue, whereas 6‐phosphogluconate dehydrogenase is shortened on average by 11 lysine residues (Machado, Ayala, Gordillo, Revilla & Santa Maria, 1991). Here, “on average” means that the observed modifications are those that occur most frequently and are readily detectable in the isolated enzyme (e.g., detectable by semiquantitative techniques such as Western blotting), whereas the actual variation among individual enzyme molecules can be vast. Damage heterogeneity (Box 1) depends on metabolic parameters of a system and the likelihood of interaction between its elements (Gladyshev, 2013).

Box 1 1 There is a great diversity of damage types, wherein each type of a “damaging insult” may influence the structure of the target molecule differently, and the same damage type may lead to structure variance of the target molecule. For example, oxidation of a single protein molecule by ROS might occur at different and sometimes multiple sites of a protein. As damage to a macromolecule changes its structure, the structural variability among the same types of macromolecules, especially when integrated across different molecules and damage types, could be one of the best markers of the aging process. Measurements of structural variability or “noise” in large populations of macromolecules could illuminate the basis of the aging process. Although for some molecules and damage types, such measurements are beyond the scope of current technology, there is emergence of such analyses in the case of DNA methylation, mutations, gene expression, and metabolite profiling. As damaged molecules may change their properties and each cell has a slightly different set of damaged molecules, metabolic pathways may be differently affected in each cell, increasing heterogeneity among cells. This consequence of damage accumulation—the increase in intracellular variability or “expression noise”—may be measurable (Bahar et al., 2006; Somel, Khaitovich, Bahn, Paabo & Lachmann, 2006; Southworth, Owen & Kim, 2009). Thus, the key property of aging—increased intracellular heterogeneity—will affect many, if not all cells. Consistently, it has been observed that intracellular heterogeneity in mRNA expression increases with age in mouse cardiomyocytes (Bahar et al., 2006), human brain cells (Brinkmeyer‐Langford, Guan, Ji & Cai, 2016), and stimulated mouse T cells (Martinez‐Jimenez et al., 2017). Finally, suppression of the transcriptional drift has been related to lifespan extension of *C. elegans* (Rangaraju et al., 2015).

It is important to put the concept of damage accumulation in aging in a broader context, which can be illustrated by the model of the “deleteriome” (Gladyshev, 2016). The deleteriome encompasses not only molecular damage but also deleterious consequences of its accumulation, such as dysregulation of gene expression, metabolic remodeling, epigenetic drift, imbalance in the components of protein complexes, and nonoptimal composition of cell types within a tissue (Gladyshev, 2016). Being a consequence of living, deleterious events at each level of biologic organization contribute to the increased disorder of the system, and their rate of accumulation is modified by genetic, environmental, and stochastic processes. By encompassing all the events that contribute to the aging process, the deleteriome model unifies many existing theories of aging. For simplicity, however, we focus in this article on the most basic types of deleterious changes with age—global accumulation of molecular damage.

## 2 GETTING TO THE CORE OF THE MATTER—WHY DOES DAMAGE ACCUMULATE?

The same imperfectness of biologic molecules and systems that allows for variation and evolution causes erroneous and damaged molecules to accumulate, leading to other deleterious changes and to aging itself. But why cannot this damage be fully cleared? One of the aging models, the disposable soma theory, states that organisms have limited resources (e.g., energy), which they allocate to either reproduction or somatic maintenance, and the trade‐offs between them determine the optimal evolutionary fitness (Kirkwood, 1977). Allocation of resources to reproduction reduces efficiency of somatic maintenance, causing damage to accumulate. This concept has been one of the first to show the link between mechanistic and evolutionary causes of aging. Its limitation lies, however, in an assumption that damage accumulation depends only on protection and repair mechanisms and that damage clearance can be perfected if sufficient resources are available. In fact, a significant share of accumulating damage is not even detectable by cellular systems, and the protective machineries, while removing some damage, produce its other forms. These issues highlight the importance of focusing on the damage unavoidably produced as a consequence of living, as opposed to considering resource allocation. Mechanisms that deal with particular forms of damage are those that evolved to act against the most deleterious damage forms (Gladyshev, 2013). Strategies dealing with cumulative damage, which are not counteracted by specific repair mechanisms, can only be diluted by cell division. Such damage dilution is the reason aging may not apply to symmetrically dividing organisms. It is also the reason it applies to organisms with differentiated, nonrenewable cells.

Symmetric distribution of damage may also slow down accumulation of mild, intracellular damage that gives populations of fast dividing cells a semblance of immortality. Accumulation of deleterious changes may be counteracted by damage dilution, especially when cells divide very rapidly and the damage they acquire is relatively mild (Clegg, Dyson & Kreft, 2014). When damage becomes difficult to cope with (dilute, repair or remove), for example under stress, it becomes easier to attach some of the damage to a certain cellular structure and to segregate it asymmetrically during cell division so that one daughter cell would inherit much of the damage burden, while the other would be depleted of it. In mammalian cells, misfolded and damaged proteins localize in juxtanuclear quality compartment (JUNQ) forming an aggregate‐like structure (Kaganovich, Kopito & Frydman, 2008). Dispersion of JUNQ into smaller aggregates and free cytoplasmic proteins is inhibited by the surrounding net of the vimentin cytoskeleton (Johnston, Ward & Kopito, 1998; Lin et al., 2016). At least some mammalian cell types show asymmetric distribution of JUNQ (Ogrodnik et al., 2014) and damaged mitochondria (Katajisto et al., 2015). Although most of asymmetric distribution phenomena in mammalian cells have been discovered in immortalized cells (Ogrodnik et al., 2014; Rujano et al., 2006), stem cells also show an asymmetric pattern of damage distribution (Bufalino, DeVeale & van der Kooy, 2013; Katajisto et al., 2015; Rujano et al., 2006). Interestingly, the accumulation of intracellular molecular damage has been suggested to be one of the main drivers of stem cell aging, upstream of epigenetic alterations, and aberrant proliferation/differentiation (Ermolaeva, Neri, Ori & Rudolph, 2018). Moreover, stem cells during differentiation‐related cell division transmit more damaged material to the daughter cells, which are destined to further differentiation and loss of stemness (Katajisto et al., 2015; Rujano et al., 2006). It is consistent with the findings that cells asymmetrically receiving more damaged material are less viable (Ogrodnik et al., 2014). The strategy of damage dilution is available, however, only to dividing cells. This strategy has limitations in organisms that possess advanced structures: highly specialized cells such as neurons, myotubes, or cardiomyocytes, which are largely postmitotic.

While all cellular macromolecules are under a constant attack of damaging insults, only some will show detectable patterns of damage buildup. The great majority of damaged macromolecules will undergo repair or become degraded. This is most common for short‐lived, easily accessible molecules such as monomeric cytoplasmic proteins and nonstructural RNA. The opposite side of the macromolecular “half‐life” scale is represented by the long‐lived macromolecules, such as DNA of postmitotic cells or proteins in inaccessible complexes (Toyama & Hetzer, 2013). For example, proteins of neuronal nuclear pore complexes that mediate trafficking in and out of the nucleus have been shown to last throughout the whole lifespan of a rat (Savas, Toyama, Xu, Yates & Hetzer, 2012). Similar longevity has been identified for some histone variants (e.g., H3.1 and H3.2), extracellular proteins (collagens and myelin proteins), and enzymes (e.g., SOD1 and ASRGL1) in the rat central nervous system (Crisp et al., 2015; Toyama et al., 2013). Weak selection and energetically high cost (or just impossibility in the case of DNA) of macromolecule replacement determine their longevity (Truscott et al., 2016). Decreasing half‐life or increasing the damage accumulation rate of such macromolecules may lead to the early onset of age‐related diseases. For example, when superoxide dismutase (SOD) gene that produces a long‐lived protein acquires a mutation (G93A), the resulting structural modification decreases its stability and half‐life (Crisp et al., 2015) causing a central nervous system disease—amyotrophic lateral sclerosis (ALS). Brain is not the only source of long‐lived, steadily accumulating damaged macromolecules. The lens serves as another well‐characterized example. The epithelial cells on the periphery of the lens lay down fiber cells that live as long as the organism does. Fiber cells in the core of a lens are produced during embryonic development and remain throughout the individual's lifespan (Sharma & Santhoshkumar, 2009). Fiber cells are deprived of organelles including the nucleus but are packed with long‐lived, water‐soluble proteins (crystallins), which enable the lens’ transparency and maintenance of the index of refraction. These proteins accumulate age‐related modifications, with the most common being oxidation, deamidation, truncation, glycation, and alkylation with concomitant increase in water‐insoluble protein fraction and appearance of big aggregates (Sharma & Santhoshkumar, 2009). Older fiber cells (closer to the core of the lens) show higher burden of damage, including water‐insoluble protein species and aggregates (Hains & Truscott, 2007; McFall‐Ngai, Ding, Takemoto & Horwitz, 1985). Similarly, extracellular macromolecules of connective tissue have a low repair/replace capacity and are known for their extreme half‐lives (Toyama & Hetzer, 2013; Verzijl et al., 2000). These are just a few examples of well‐known, long‐lived, damage‐accumulating proteins with important functions.

Shorter lifespan of other macromolecules does not make them invulnerable to damage accumulation or irrelevant to the aging process. For example, the accumulation of age‐related changes in long‐lived macromolecules may increase the rate of damage accumulation of even the short‐lived ones. Alongside DNA and proteins, carbohydrates and lipids can spread damage, for example, by forming aggregates (e.g., lipofuscin) that can trap other macromolecules (Terman & Brunk, 2004). Because the majority of biologic molecules have the potential to produce damage, the rate of damage accumulation always exceeds the repair/degradation capacity. It is also known that efficiency of the damage “repair or removal” machinery decreases with aging (Kaushik & Cuervo, 2015) and that mutations in the associated genes lead to accelerated aging (Liao & Kennedy, 2014).

What alleviates damaging modifications impairing macromolecule function, counteracting deleterious processes, is a poorly understood “stability” of macromolecules. This term broadly encompasses higher resistance to denaturation, aggregation, and other structure‐altering modifications while in contact with chemicals and under various stress stimuli (e.g., high temperature or oxidative stress). The mysteriously high resistance of proteins and other classes of macromolecules has been observed in long‐lived organisms (Pickering, Lehr, Kohler, Han & Miller, 2015; Salmon et al., 2009; Treaster et al., 2014) and in laboratory model organisms with increased lifespan (David et al., 2010; Depuydt, Shanmugam, Rasulova, Dhondt & Braeckman, 2016). In vivo, higher stability can be obtained through higher fidelity of macromolecule production (e.g., lower frequency of errors during DNA replication or protein translation) as well as through higher availability of stabilizing compounds and chaperone macromolecules.

Macromolecular damage primarily affects nonrenewable cells whose replacement is impossible or energetically costly (such as neurons or cardiomyocytes). However, while damage accumulation relates to these long‐lived cells, short‐lived cells are also relevant to the aging process (Box 2). These cells, in addition to the rise of damage during their life cycle, have to deal with the damage burden inherited from their progenitor cells. Erythrocytes isolated from old mice are burdened with certain amount of damage just after they differentiate from hematopoietic stem cells (HSCs) (Kumar & Rizvi, 2014). These erythrocytes live shorter and accumulate damage faster than the erythrocytes isolated from young mice (Kumar & Rizvi, 2014). When the damage burden in HSCs is too high, they might stop to differentiate, entering the state of cellular senescence or undergoing apoptosis (Wang, Lu, Sakk, Klein & Rudolph, 2014), decreasing concomitantly the number of functional blood cells. Similarly, transmission of mutations and other types of genomic damage from stem cells impairs their progenitors’ function that has been related to age‐related diseases and cancer risk (Adams, Jasper & Rudolph, 2015).

Box 2 1 The process of damage accumulation in short‐lived cells can be illustrated using an uncommon example of aging—erythrocytes. Red blood cells are deprived of the nucleus, mitochondria, and the majority of organelles. After they differentiate from myeloid progenitor cells, they are left only with the basic repair and removal machinery while lacking capacity for protein production. In these cells, ROS are generated, instead of mitochondria, by failed binding of oxygen to heme which can generate superoxide. Oxidative stress and other types of damaging insults impair function of red blood cell enzymes (Jindal, Ai, Gascard, Horton & Cohen, 1996), damage cell membrane proteins (Ando, Beppu, Kikugawa, Nagai & Horiuchi, 1999) and lipids (Kumar & Rizvi, 2014), and cause accumulation of an autofluorescent oxidation end product—lipofuscin (Khandelwal & Saxena, 2007; el‐Rahman, Hammouda & Fakeir, 1995). The exponential increase in lipofuscin‐related accumulation is easily observable during the short life of an erythrocyte (Khandelwal & Saxena, 2007). Finally, damage to the cell membrane is recognized by macrophages, which phagocytize the red blood cells. Rapid aging of erythrocytes shows how damage accumulation affects a unit of limited repair/removal capacity and could serve in future studies aiming to better understand this aspect of the aging process.

In summary, macromolecules are susceptible to modifications that are accumulated during cellular and organismal aging. Modifications/damage types are greatly heterogeneous and numerous. Organisms evolve strategies to deal with those that directly impact their fitness but are unable to counteract all damage types. Certain damage types may differ depending on environmental and metabolic conditions, but the cumulative damage increases with age. Cumulative molecular damage, a part of the deleteriome, is the most fundamental, defining feature of aging.

## 3 CHALLENGES WITH EXPERIMENTAL/PRACTICAL APPLICATIONS OF DAMAGE ACCUMULATION IDEAS TO AGING RESEARCH

The idea that damage is a critical factor in aging dominated the field for some time, as correlative evidence on the occurrence of damage and its role in aging is overwhelming (Gladyshev, 2013; Kirkwood, 2005; Zimniak, 2008). However, in recent years, their prominence was challenged by the theories of programmed and hyperfunction aging. These theories postulate that aging occurs due to an “internal program” (programmed aging) or its “wasteful and aimless continuation” (hyperfunction) (Blagosklonny, 2013). However, neither programmed aging is consistent with the evolutionary logic nor organisms in any species have been observed that disrupted such a program in response to mutations. If all other genetic programs can be disrupted by mutations, why not the aging program? The concept of hyperfunction considers damage as unimportant in causing aging. Instead, it postulates that overactivity of master metabolism and growth regulators such as mTOR are the main drivers of aging (Blagosklonny, 2012; Gems & de la Guardia, 2013). It was proposed that the evidence behind these theories lies in the reduced activity of pathways that prolong lifespan. For instance, rapamycin given to mice late in their lives causes the reduction in TOR signaling and leads to lifespan extension (Harrison et al., 2009). However, it is also known that the diseases characterized by TOR hyperactivation do not necessarily accelerate aging (Caban, Khan, Hasbani & Crino, 2017; Henske & McCormack, 2012). This may be rationalized by the fact that nutrient signaling pathways impact on the rate of aging, but they are not directly causal for aging. Interestingly, the majority of known progeroid mouse models and human diseases of accelerated aging are related to genes that are responsible for repair or removal of damage (Liao & Kennedy, 2014). On the other hand, evidence supporting the role of damage accumulation in aging (Gladyshev, 2013; Kirkwood, 2005; Zimniak, 2008) is often neglected as there are certain issues that made it difficult to test these ideas experimentally and apply to life‐extending therapies.

**Figure 2:**
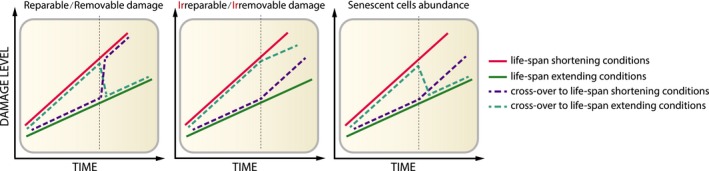
**Accumulation patterns of damage types and senescent cells in an organism subjected to lifespan‐modifying interventions.** (left graph) Repairable damage, such as oxidative damage, exhibits a full reversal of the phenotype so that an intervention extending the lifespan sharply decreases damage level, while an intervention decreasing lifespan elevates damage. (middle graph) Level of irreparable damage, such as lipofuscin, is not changed by lifespan‐modulating interventions, in contrast to the rate of its accumulation, demonstrating the importance of life history on irreparable damage accumulation. (right graph) Markers of cell senescence change independently of damage markers. Upon entering a lifespan‐extending treatment, an organism shows a long‐lasting decrease in senescence markers. Changing the conditions to lifespan shortening does not affect the current status of senescence markers but might impact on the rate of senescent cell accumulation

**Figure 3:**
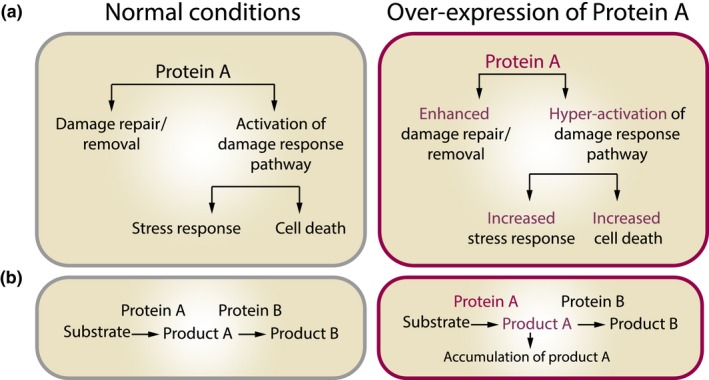
**Unexpected effects of overexpression of proteins involved in macromolecular damage repair and/or removal.** Overexpression of a protein may yield unexpected results, which are related to (a) multifunctionality of proteins and (b) accumulation of products of the enzymatic reaction carried by the overexpressed protein

1. *Particular damage types do not represent aging*. Some studies have suggested that the observed patterns of damage accumulation are inconsistent with the primacy of damage accumulation in certain models of longevity. This especially relates to oxidative damage, which has been shown to be elevated in some long‐lived animals such as the naked mole rat (Andziak et al., 2006) and long‐lived mouse mutants (Lapointe, Stepanyan, Bigras & Hekimi, 2009). Importantly, overexpression of antioxidant enzymes in mice led to mixed results, with some interventions leading to an increase in maximum and average lifespan (Mitsui et al., 2002; Schriner et al., 2005) or an increase only in earlier part of murine life (Perez et al., 2011), but most did not affect the lifespan (Jang et al., 2009; Perez, Bokov, et al., 2009; Perez, Van Remmen, et al., 2009). The ostensible discrepancy between age‐related damage accumulation and the lack of relevance of oxidative damage for the lifespan extension was also examined using *Drosophila melanogaster* as a model of aging (Jacobson et al., 2010). In this system, conditions promoting longevity decreased oxidative damage (Figure 2, left graph), whereas conditions shortening lifespan resulted in an increase in oxidative damage, regardless of animal's life history. This finding suggested that oxidative damage can be repaired and/or removed and that it does not necessarily represent the type of damage that underlies aging in this species. In contrast, the levels of advanced glycation end products (AGEs) and presumably other final products of damage propagation, such as lipofuscin, did not diminish upon switching to lifespan‐increasing conditions (Figure 2, middle graph). Instead, flies kept under such conditions slowed down the rate of AGE accumulation and those subjected to lifespan‐decreasing conditions showed an increased rate of AGE accumulation. The change in the damage accumulation rate without affecting the levels of damage already existing, in the form of AGEs, points to its importance for aging. Using a similar experimental approach, the relevance of particular types of damage can be tested; that is, introducing life‐extending treatments would then affect the damage accumulation rate. However, the relative contribution of this damage to the aging process would still be unclear. Perhaps, the damage that best correlates with aging may be considered a biomarker of aging. For example, in postmitotic cells, there is a steady accumulation of lipofuscin and mutations with age (Couve, Osorio & Schmachtenberg, 2012; Hoang et al., 2016; Lodato et al., 2018; Porta, Llesuy, Monserrat, Benavides & Travacio, 1995; Terman & Brunk, 2004).
  Similar conclusions were obtained in experiments that tested other individual damage types. For example, DNA mutations are known to increase with age, their increased numbers (e.g., in response to knockout of DNA repair machinery) may accelerate aging, and their decreased numbers (e.g., in response to overexpression of DNA repair genes) may slow it down. However, quantification of age‐related mutations in *Saccharomyces cerevisiae* revealed on average only 0.4 mutations per lifespan of a cell (Kaya, Lobanov & Gladyshev, 2015). In other words, all yeast cells aged and died, whereas only some of them accumulated even a single mutation, indicating that mutations, when taken in isolation, do not cause aging. This concept may extend to other damage types, wherein these damage forms, even if not causal in isolation, may cause aging when acting together. The effect of cumulative damage on aging can be assessed by reintroducing it through diet to the organism that accumulates it during its lifetime (Lee et al., 2017). Application of this concept to budding yeast, fruit flies, and mice revealed that higher cumulative damage is associated with shorter lifespan. Thus, cumulative damage may cause aging even if its individual components may not.
2. *Damage repair/removal proteins may have more than one function*. An increase in the expression and activity of repair proteins does not only facilitate the repair process but also informs the cell about the severity of the damage form. Such signaling can lead to stress response, cell cycle arrest, senescence, or apoptosis. Activity of the DNA repair protein PARP correlates with longevity of mammals (Grube & Burkle, 1992), but paradoxically overexpression of human PARP gene in mouse leads to a significant decrease in lifespan (Mangerich et al., 2010). This might be due to the multitude of additional roles PARP plays, such as an apoptosis initiator and a metabolic, epigenetic, and transcription regulator (Bai & Canto, 2012; Posavec Marjanovic, Crawford & Ahel, 2017). Overall, overexpression of this multifunctional protein leads to confounding outcomes‐ enhanced repair yet increased cell death (Figure 3a).
3. *Repair/removal of macromolecular damage requires cooperation of many proteins*. To be efficient, this process typically requires separate systems for damage recognition, signaling, attachment, and finally for repair/removal. Each of these systems contains dozens of elements which can do its part only if upstream elements are functional. Such systems have relatively low redundancy so that mutations in a single subunit of a repair/removal pathway can result in a strong phenotype, often related to shorted lifespan. Finally, overexpression of only a single enzyme in a repair pathway can lead to the accumulation of its product that might not be metabolized by non‐overexpressed downstream component and can be toxic (Figure 3b). For example, failure of lifespan extension in mice overexpressing superoxide dismutase enzymes (Perez, Bokov, et al., 2009; Perez, Van Remmen, et al., 2009) might be due to the accumulation of its product, hydrogen peroxide, as it was observed in other models of SOD overexpression (Jivabhai Patel et al., 2015; Peled‐Kamar et al., 1997; Usui et al., 2011). This obstacle could be overcome by overexpression of multiple proteins that repair/remove damage. Such manipulations are already possible; for example, simultaneous overexpression of up to four genes in *C. elegans* showed an additive effect on lifespan extension (Sagi & Kim, 2012).
4. *Unexpected roles of damaging stimuli and damage detection systems*. Attempts to manipulate damage levels and protection systems have shown that increasing the capacity of damage detection, defense, repair, or removal systems can give unexpected and sometimes opposite to the expected results. For example, cellular protection systems can receive a boosting effect after a low dose of harmful insult, a phenomenon called hormesis. The effects of hormesis can be seen in cases of low‐grade stress such as physical exercise or lower calorie intake increasing various organismal resistances (Mattson, 2008). In the context of damage accumulation, low‐grade stress will cause an increase of repairable/removable damage that is detected by the system and may therefore upregulate global defense systems. Accordingly, *Mclk*+/− mice that have increased oxidative stress and diminished irreversible cytoplasmic damage show increased maximum lifespan (Lapointe & Hekimi, 2010; Lapointe et al., 2009). Interestingly, the mortality curve of *Mclk*+/− mice compared to control shows a decrease in the rate of aging (Lapointe et al., 2009), suggesting an effect on the damage accumulation rate. Long‐term beneficial effects of hormesis are currently known for oxidative damage, and it is unlikely that an increase in irreparable/irremovable damage elicits a similar, beneficial response. This kind of damage due to its heterogeneity is difficult to detect and, unlike oxidative damage, might not reflect the type of stress stimuli that affects the cell.

Another complication that has been described for oxidative stress is its role in cell signaling. Some ROS (particularly superoxide and hydrogen peroxide) are involved in intracellular signaling of MAP kinases (McCubrey, Lahair & Franklin, 2006) and mitogenic RAS pathways (Irani et al., 1997; Mitsushita, Lambeth & Kamata, 2004), inflammasome activation (Dostert et al., 2008; Petrilli et al., 2007), wound detection, and healing (Han et al., 2014; Niethammer, Grabher, Look & Mitchison, 2009), among others. Functionality of these processes can be altered via antioxidants (Dostert et al., 2008; Han et al., 2014; Irani et al., 1997; Petrilli et al., 2007) or manipulations in ROS scavenging systems (Dostert et al., 2008; Han et al., 2014). It is possible then that interventions into the redox system aimed at decreasing damage generation might additionally affect one or more of the above‐listed processes. Similarly, proteasomes that are responsible for degradation of damaged proteins are also used to produce peptides presented on the cell surface to major histocompatibility complex class II (Lehner & Cresswell, 1996) or to regulate cell cycle progression (King, Deshaies, Peters & Kirschner, 1996). Therefore, interventions into the activity of proteasomal system aimed at facilitating damage removal could result in additional effects obscuring the experimental outcome.

Overall, current approaches to extend lifespan directly by slowing down damage accumulation have given inconsistent results. This is because we do not know what types of damage (or to what macromolecules) play major roles in aging. From an evolutionary standpoint, many damage types are expected to contribute, and their rates of increase may be roughly synchronized. So, targeting any single form may be futile. In light of these considerations, it might seem impossible to achieve a consistently beneficial effect on lifespan via decreased cellular damage, when a particular process or even several processes are targeted. Instead, more successful interventions into the damage accumulation process can be executed through manipulations of master regulators of metabolism, growth, and maintenance. Examples of such regulators are IGF1 and mTOR, but perhaps many remain to be discovered. Changing the activity of these pathways may lead to global changes in cell/organism metabolic state, and in the context of cellular aging, it will result in a substantially different set of damage forms produced, thereby altering the overall damage accumulation rate through slowing down metabolism and upregulation of cellular defense and maintenance systems. For instance, inhibition of mTOR decreases ROS production and accumulation of intracellular damage, simultaneously increasing the expression of proteins involved in autophagy and proteasome degradation systems (Lelegren, Liu, Ross, Tardif & Salmon, 2016; Martinez‐Cisuelo et al., 2016; Miwa et al., 2016; Yun et al., 2016). mTOR inhibition also increases the fidelity of translation and quality of newly synthesized proteins (Conn & Qian, 2013). It is not surprising then that the greatest lifespan extension has been achieved through genetic and pharmacological manipulations of aforementioned pathways. To overcome limitations related to the enhancement of protection against damage, interventions that target many forms of the already accumulated damage could be implemented. A cellular phenomenon characterized by highly elevated levels of multiple types of damage is cellular senescence.

## 4 DAMAGED CELLS THAT DO NOT DIE—THE ROLE OF CELLULAR SENESCENCE

### 4.1 What is cellular senescence and how is it related to damage accumulation?

A cellular phenomenon described for the first time in 1965, cellular senescence (Hayflick, 1965), was named so due to its resemblance to the organismal aging process. It has been observed that cells from primary cultures decelerate their division rate over time to permanently stop dividing within few weeks of culture. This phenomenon is known as replicative senescence. Cellular senescence, however, may be accelerated by changing the environment to that characterized by increased damage generation, for example, by elevating the concentration of oxygen or applying various toxins (Cristofalo, Lorenzini, Allen, Torres & Tresini, 2004; Parrinello et al., 2003; von Zglinicki, 2002). On the other hand, factors that decelerate damage accumulation, such as inhibitors of mTOR, delay the appearance of senescence phenotypes (Demidenko et al., 2009). A cell can enter damage‐induced cellular senescence either by gradual accumulation of damage in replicative aging or by a sudden outburst of damage induced by radiation or chemotherapeutics. Therefore, senescent cells often show high levels of various forms of damage and resemble chronologically aged cells observed in an old organism. However, senescence is not the final state of chronological aging of cells. While the cell stops dividing upon entering senescence, it keeps accumulating damage in the process that can last months or even years ending in cell death (Fumagalli, Rossiello, Mondello & d'Adda di Fagagna, 2014; Sitte, Huber, et al., 2000; von Zglinicki, Nilsson, Docke & Brunk, 1995).

Despite attempts to define one marker uniquely specific to senescent cells, it is generally accepted by the community that identification of senescent cells requires a multimarker approach, particularly because some frequently used markers are not exclusive to senescent cells. Cellular senescence is considered a temporally regulated and dynamic process with its complex phenotypes balancing both pathological and physiological contexts (Childs, Durik, Baker & van Deursen, 2015; Hoare et al., 2016). The heterogeneity of senescent phenotypes is even greater when different senescence inducers or divergent cell types are taken into consideration (Hernandez‐Segura et al., 2017). Moreover, the heterogeneity resulting from a type of an insult used to induce senescence also impacts on what markers are expressed by senescent cells (as reviewed in (Munoz‐Espin & Serrano, 2014)). Finally, as the field is still in its infancy in understanding senescence in vivo, the majority of data discussed here come from in vitro experiments.

**Figure 4:**
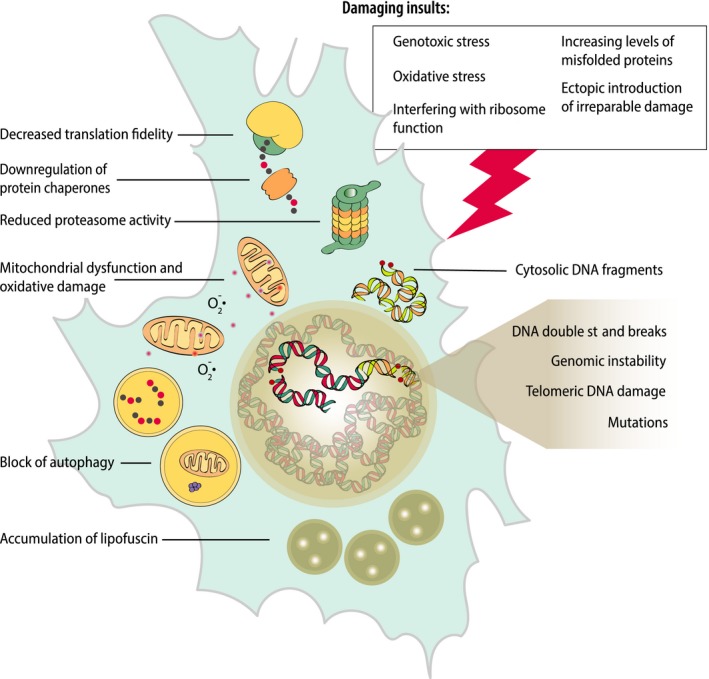
**A summary of damage types known to accumulate in senescent cells and damage‐dependent triggers of cellular senescence**. Cells induced to senesce by damaging insults exhibit higher than healthy cells basal levels of damage and generate damage at a higher rate

Various forms of molecular damage, shown to be increased in senescent cells, include DNA damage, aggregates, and oxidative damage (Figure 4). Other markers of aging detected in both aging organisms and senescent cells include dysfunctional mitochondria, decreased autophagy flux, epigenetic changes, short telomeres, and inflammation.

### 4.2 Importance of DNA damage in cellular senescence

DNA damage types in cell senescence include oxidative modifications, single‐ and double‐strand breaks (DSBs), as well as mutations, both in vitro and in vivo (d'Adda di Fagagna, 2008; Busuttil, Rubio, Dolle, Campisi & Vijg, 2003). DSBs exhibit the highest cytotoxicity among DNA damage forms and can propagate damage leading to mutations and genome instability (Li, Mitchell & Hasty, 2008). DNA double‐strand breaks are constantly generated by environmental factors and changes in DNA topology (e.g., occurring during replication or transcription). In senescent cells, the repair of DSBs through DNA end joining is less efficient and is seen in decreased fidelity, leading to the accelerated accumulation of sequence errors and genomic instability (Seluanov, Mittelman, Pereira‐Smith, Wilson & Gorbunova, 2004). The accumulation of DSBs in an age‐dependent fashion has been reported in cells of multiple mammalian tissues (Jurk et al., 2012, 2014; Rube et al., 2011; Wang et al., 2009). To directly study the role of DSBs in senescence induction in vivo, an adenovirus‐based mouse model inducing DSBs through expression of the transgenic restriction enzyme SacI is often used (White et al., 2015). Induction of DSBs as a sufficient cellular senescence trigger has been shown (d'Adda di Fagagna, 2008). The study by White and colleagues was the first to demonstrate that the induction of DSBs can be a driver of hepatocyte senescence in vivo. A month after the induction of DSBs, an increase in the markers of senescence (karyomegaly, polyploidy, *p16* and *p21* expression) and accelerated liver aging (infiltration of immune cells and apoptosis) were observed.

In contrast to DSBs in nontelomeric regions of chromosomes, breaks within telomeres (telomere‐associated foci, TAF) are considered persistent and irreparable (Hewitt et al., 2012). Irreparability of TAF is explained mainly through inhibition of DNA repair enzymes by telomere‐binding proteins that prevents chromosomal fusion caused by telomere shortening (and executed by nonhomologous end joining [NHEJ] pathway; Bae & Baumann, 2007; Fumagalli et al., 2012). Both stress‐induced senescence (H_2_O_2_, X‐ray, and genotoxic) and oncogene‐induced senescence (through DNA replication stress and replication fork stalling) have been demonstrated to cause the formation of repairable DSBs at nontelomeric regions and irreparable DSB at telomeres (Hewitt et al., 2012). Accumulation of TAF‐burdened cells has been found in vivo in multiple tissues as a function of age (Hewitt et al., 2012; Jurk et al., 2014) and disease (Ogrodnik et al., 2017; Roos et al., 2016; Schafer et al., 2017).

Recently, discovery of nuclear DNA in the cytoplasm of senescent cells added another functional consequence of DNA damage. Cytosolic chromatin is found in the form of cytoplasmic chromatic fragments (CCF) as a consequence of X‐ray and genotoxic insults (e.g., with etoposide), as well as in oncogene‐induced senescence and replicative senescence. The presence of damaged (positive for markers of DSBs) chromatin is recognized by cytosolic DNA‐sensing pathway and activates a proinflammatory response (Dou et al., 2017; Ivanov et al., 2013).

### 4.3 Importance of protein and lipid damage in senescent cells

As most proteins and lipids are considered relatively short‐lived, the level of their damage depends on the damage accumulation rate and the opposing degradation/repair efficiency. Quality of proteins is assured by the translation machinery and the activity of helper proteins (including chaperones), while degradation is governed by autophagy and proteasome functions. All these core damage turnover mechanisms have been found to be affected in senescent cells.

One of the most important features of cellular senescence, the senescence‐associated secretory phenotype (SASP) (Coppe et al., 2008) requires an increased protein synthesis rate, and this is achieved by hyperactivation of mTOR in senescent cells (Correia‐Melo et al., 2016; Herranz et al., 2015; Laberge et al., 2015). While mTOR is responsible for the global alteration of the translation rate, increased protein synthesis affects not only secretory factors but all synthesized proteins (Deschenes‐Simard et al., 2013; Herranz et al., 2015; Salama, Sadaie, Hoare & Narita, 2014). Hyperactivation of mTOR increases the speed of ribosomal elongation, but, at the same time, decreases translation fidelity, resulting in the increased rate of errors, thereby generating more proteins of lower quality (Conn & Qian, 2013). Another factor that potentially increases protein translation in senescence is increased ribosome biogenesis (Nishimura et al., 2015). Interfering with the translation machinery either through inhibition of mTOR (Demidenko et al., 2009; Laberge et al., 2015; Walters, Deneka‐Hannemann & Cox, 2016) or through decreased ribosome biogenesis (Nishimura et al., 2015) attenuates but does not reverse (Laberge et al., 2015) cellular senescence. Finally, activation of mTOR under nutrient‐high conditions (Guo et al., 2014; Nakano et al., 2013; Xiong et al., 2014; Zhang et al., 2012) or perturbation of ribosome biogenesis drives cells into senescence (Nishimura et al., 2015), possibly through accumulation of damage resulting from lower quality of translation. Higher production and lower quality of proteins in senescent cells also impose a need for increased protein degradation, which is, however, not met by degradation machineries (Deschenes‐Simard et al., 2013).

Proteasomes are highly active in proliferating cells, removing not only damaged proteins but also those whose degradation is crucial for cell cycle progression. In cellular senescence, the profile of ubiquitinated substrates shifts significantly. Senescent cells show a different pattern of ubiquitination of proteins that are involved in translation, DNA repair, chaperone, and mitochondria‐related machineries, which are closely linked to initiation and sustainment of cellular senescence (Bengsch et al., 2015; Deschenes‐Simard, Lessard, Gaumont‐Leclerc, Bardeesy & Ferbeyre, 2014). Not only the specific proteasome substrates but the total amount of ubiquitination substrates and the activity of proteasomes itself change. Stress‐induced senescence leads to the reduction in proteasome activity and an increased pool of ubiquitinated proteins (Gamerdinger et al., 2009; Pan, Short, Goff & Dice, 1993; Sitte, Merker, Grune & von Zglinicki, 2001). That might be a direct cause of severe (e.g., during stress‐induced senescence) and sustained (e.g., during replicative senescence) oxidative stress (Shang & Taylor, 2011) as both ROS and ROS‐induced damage have been shown to inhibit 20S and 26S parts of the proteasome (Gracanin et al., 2011; Powell et al., 2005; Reinheckel et al., 1998; Sitte, Huber, et al., 2000) or other forms of intracellular damage.

Macroautophagy has been well studied in the context of cellular senescence (Grasso & Vaccaro, 2014; Narita, 2010). The relationship between autophagy and cellular senescence is, however, complex (Kwon, Kim, Jeoung, Kim & Kang, 2017), and it varies in relation to the type of damage inducer; for example, it can differ between oncogene‐ and oxidative stress‐induced senescence (Tai et al., 2017; Young et al., 2009). mTOR is one of the main inhibitors of autophagy, but counterintuitively, even with hyperactive mTOR, senescent cells show increased levels of components of macroautophagy (Gamerdinger et al., 2009; Young et al., 2009). One possible explanation is that senescent cells display unique spatiotemporal separation of the mTOR complex from the macroautophagy machinery (Narita et al., 2011; Young & Narita, 2011). Macroautophagy is crucial for the transition to cellular senescence, and its inhibition leads to alleviation of the senescent phenotype (Gamerdinger et al., 2009; Young et al., 2009). Moreover, efficiency of macroautophagy has been directly linked to SASP (Dorr et al., 2013; Narita et al., 2011) and telomere dysfunction (Mar, Debnath & Stohr, 2015). Considering the impairment of chaperone‐mediated autophagy and decreased proteasomal degradation, macroautophagy appears to be the most likely player in the senescence‐associated increase of protein degradation (Deschenes‐Simard et al., 2014). One of the most distinguishable, albeit not essential, features of cellular senescence‐increased activity of senescence‐associated β‐galactosidase (SA‐β‐Gal) (Dimri et al., 1995) arises from increased levels of lysosomes, the final terminators of macroautophagy‐related degradation (Lee et al., 2006). Alongside elevated lysosomal content, an increased activity of lysosomal enzymes has been demonstrated (Knas et al., 2012; Lee et al., 2006). However, despite the increase in components of autophagic machinery, the overall flux decreases (Tai et al., 2017). Cellular senescence induced by oxidative stress impairs autophagy flux, shown as decreased capacity of lysosomes in quenching the green fluorescence ofmRFP‐GFP‐LC3 and decreased degradation of p62 (Tai et al., 2017). Finally, a higher content of lysosomes and other autophagy vesicles in senescent cells may stand behind another feature of cellular senescence‐increased granularity (von Zglinicki, 2002). It is possible then that the observed increase in lysosomal content and cell granularity are due to partial inability to degrade lysosomal content—heavily cross‐linked proteins and damaged lipids that are retained in the lysosomes of senescent cells.

Another feature affecting protein damage levels in cellular senescence is a downregulation of cellular chaperones, mainly Hsp90, Hsp70, and small heat‐shock protein families (Deschenes‐Simard et al., 2013; Gabai, Yaglom, Waldman & Sherman, 2009; Gamerdinger et al., 2009; O'Callaghan‐Sunol, Gabai & Sherman, 2007; Yaglom, Gabai & Sherman, 2007). Chaperones are necessary not only for proper folding of newly synthesized and already existing proteins, but also for the degradation of misfolded proteins in chaperone‐mediated autophagy (CMA). Consistent with the aberrations in chaperone protein levels, CMA has been found to be downregulated in senescent fibroblasts (Cuervo & Dice, 2000; Dice, 1982; Okada & Dice, 1984). Decreased chaperone levels not only negatively affect quality of proteins, but also increase the probability of aggregate formation (Duncan, Cheetham, Chapple & van der Spuy, 2015; Paul & Mahanta, 2014). Finally, interventions that are known to increase the level of misfolded proteins and intracellular aggregates have been found to induce cellular senescence. These include knockout of Hsp72 (Gabai et al., 2009), downregulation of Hsp27 (O'Callaghan‐Sunol et al., 2007), and drug‐induced inactivation or knockout of Hsp90 (Han et al., 2017; Sarangi, Paithankar, Kumar, Subramaniam & Sreedhar, 2012).

Similar to aging erythrocytes (Khandelwal & Saxena, 2007) (Box 2) and postmitotic tissues (Jung, Bader & Grune, 2007), stress‐ and replication‐induced senescent cells accumulate lipofuscin (Evangelou et al., 2017; Georgakopoulou et al., 2013; Sitte et al., 2001; von Zglinicki, 2002). In senescent cells, lipofuscin probably originates from macromolecules that have been oxidized by primary (senescence‐inducing agents) or secondary (dysfunction of mitochondria in late senescence) stress (Sitte et al., 2001). Lipofuscin resides primary in lysosomes (Tsuchihashi et al., 2015) and, if not separated by lysosomal membrane, further inhibits the already impaired proteasome system (Sitte, Merker, von Zglinicki & Grune, 2000). Furthermore, treatment of cells with AGEs (Mosieniak et al., 2012) or lipofuscin (von Zglinicki et al., 1995) has been shown to be sufficient to induce cellular senescence.

An outstanding question remains as to whether proteostatic stress, high abundance of lower quality, potentially toxic proteins, and the dysfunction of protein degradation machinery in senescent cells lead to the formation of aggregates and/or sequestration and degradation compartments known as JUNQ or aggresome (JUNQ is defined as JuxtaNuclear Quality Control Compartment, while aggresome is less precisely defined and comprises JUNQ and insoluble types of aggregate such as IPOD). Although neither the presence nor a possible role of JUNQ has been reported for senescent cells, there have been some observations, suggesting it may be the case. Smaller aggregates are transported along the microtubules to their final destination in JUNQ in the HDAC6‐dependent manner (Kawaguchi et al., 2003). Consistently, upregulation of HDAC6 has been observed in senescence (Akare et al., 2006; Edmond, Brambilla, Brambilla, Gazzeri & Eymin, 2011). Filaments of vimentin cytoskeleton are known to form a cagelike structure separating the aggregate from the rest of the cytoplasm (Johnston et al., 1998; Ogrodnik et al., 2014) and might be involved in capturing smaller aggregates to JUNQ (Lin et al., 2016; Ogrodnik et al., 2014). Repositioning or increased expression of vimentin has been reported for senescent cells (Carey, Knowell, Chinaranagari & Chaudhary, 2013; Litwiniec, Gackowska, Helmin‐Basa, Zuryn & Grzanka, 2013; Nishio & Inoue, 2005; Nishio, Inoue, Qiao, Kondo & Mimura, 2001), while overproduction of vimentin has been shown to induce senescent‐like morphology of fibroblasts (Nishio et al., 2001). The appearance of JUNQ‐like structures has been noted as an effect of X‐ray irradiation (Salemi, Almawi, Lefebvre & Schild‐Poulter, 2014) and in oncogene‐induced senescence (Narita et al., 2011), but never described as a hallmark of cellular senescence.

Lipofuscin is a nondegradable product of protein and lipid oxidation shown to accumulate in postmitotic and senescent cells (Sitte et al., 2001). An outstanding question—whether these cells accumulate lipofuscin predominantly over proliferating cells due to inability to dilute or completely dispose it during cell division—remains to be addressed. The concept of disrupted damage dilution through cell division system could also explain why one type of cells accumulate damage faster upon induction of senescence (cells are unable to divide and dilute damage) than in a nonsenescent state (able to divide and dilute damage).

Interestingly, factors secreted by senescent cells also exhibit a paracrine effect on neighboring cells (Nelson et al., 2012). The so‐called bystander effect drives DNA damage and lipid oxidation in cells in close proximity to senescent cells, which has been demonstrated both in vivo and in vitro (Acosta et al., 2013; Nelson et al., 2012). The bystander effect that induces intracellular damage accumulation in neighboring cells and chronic inflammation leads to tissue dysfunction (Acosta et al., 2013). While the role of the bystander effect has been related to the risk of age‐related pathologies, how it affects the rate of aging remains unclear.

In summary, a cell can enter senescence through multiple damage‐related processes, for example, due to a burst of damage‐inducing stimuli (e.g., X‐ray) or the steady accumulation of damage in replicative senescence. The second process can be accelerated through external (e.g., oxidative stress from higher O_2_ concentration) or internal (e.g., lower fidelity of translation) stimuli. On the other hand, damage in senescent cells not only is a trigger of senescence but also accumulates at an accelerated rate. The accelerated damage accumulation in senescent cells results from decreased fidelity of cell housekeeping processes (e.g., translation or DNA repair), increased levels of internal damaging stimuli (e.g., ROS), and overburdened quality control and degradation systems that are compromised in senescence (Figure 4). All this proves how important the damage accumulation process is for the induction and progression of cellular senescence and suggests that what distinguishes senescent from healthy cells both in vitro and in vivo is the exceptionally high level of cellular damage.

## 5 HOW CELLULAR SENESCENCE TIES WITH THE CONCEPT OF GLOBAL DAMAGE ACCUMULATION

The damage that accumulates with age is highly heterogeneous and can vary among the types of macromolecules, cells, and tissues. Moreover, not all types of damage will accumulate linearly with age or must be directly driving the aging process. Altogether, damage itself might be a very elusive target for lifespan‐increasing interventions. Alternative or indirect ways to decrease the damage levels and its accumulation might not always prove successful because of the complexity of damage repair and removal processes. In some cases, the consequences of damage accumulation might be easier to target than the primary cause of damage itself. In this respect, cellular senescence appears to be one of the best candidates for such interventions.

Fluctuation of a damage level upon lifespan‐extending interventions can often determine the impact of particular damage types on the rate of aging (Jacobson et al., 2010) (Figure 2). Determining the kinetics of senescence markers could help establish how the beneficial effects of life‐extending treatments are associated with senescence. A recently published study examined markers of senescence in mouse liver before and after 3‐month long dietary restriction (Ogrodnik et al., 2017). Upon short‐term DR, the number of hepatocytes stained positive for senescence markers decreases when compared to ad libitum (AL) fed mice whose tissues were collected before the initiation of DR (Ogrodnik et al., 2017) (Figure 2, right graph). Such markers include irreparable TAFs, implying that the intervention did not alleviate the senescence phenotype, but led to the actual senescent cell removal. The decrease in senescent cell abundance was accompanied by the alleviation of an aging phenotype, which in this study was the ectopic accumulation of lipid droplets in the liver. Similar kinetics has been recorded for the level of oxidative damage in flies that had their dietary intake restricted to a short period of time (Jacobson et al., 2010). These data suggest that the abundance of senescent cells, similar to findings on oxidative damage, does not depend on the life history of an animal. However, when mice had their diet regime switched back to AL feeding, both liver pathology and frequency of senescent cells remained at a low level for at least 3 months. In contrast, oxidative damage level went back to the pre‐DR level when flies have their diet regime switched back to AL feeding (Figure 2, left graph). Altogether, the level of senescent cell markers shows different kinetic patterns upon lifespan‐extending intervention than the damage markers studied by Jacobson et al. (2010): The markers of senescence decrease when lifespan is extended, which seems to be a long‐term effect, even when the beneficial intervention is halted (Figure 2, right graph).

The main definition of cellular senescence, namely “a state of irreversible cell cycle arrest,” implies “binarism” of this process; that is, a cell cannot be “partially senescent” because a partial cell cycle arrest does not exist. The binary separation between senescence and nonsenescence is considered to be defined by the level of accumulated damage, length of telomeres, or activity of oncogenes, which, when reaching a certain threshold, execute the division halt. The same part of cellular senescence definition has kept postmitotic cells largely beyond the interest of the field. Recently, however, some postmitotic cells such as neurons have been found to acquire a senescent‐like phenotype (Jurk et al., 2012). The observation of postmitotic cells exhibiting a senescent‐like phenotype suggests other possibilities for senescence to affect the aging process of an organism. First, establishment of senescence in postmitotic cells could be less binary as it is independent of the cell cycle arrest; that is, intensity of senescent phenotype could increase in these cells gradually over time. Second, it would be worth testing whether induction of senescence in post mitotic cells also accelerates damage accumulation in these cells and contributes to the development of age‐related pathologies. Finally, the spatiotemporal features of postmitotic cells such as neurons or muscle fibers may prevent them from being eliminated by immune cells. The question of whether impaired immune clearance of postmitotic senescent cells increases their senescent lifespan and allows them to drive damage accumulation of postmitotic tissues should be further addressed experimentally.

## 6 MORE QUESTIONS THAN ANSWERS—THE EFFECTS OF SENESCENT CELL CLEARANCE

Treatments ameliorating or postponing the features of cellular senescence including the SASP as the most prominent one have been known for a long time. Recently, a new class of drugs—”senolytics”, which eliminates senescent cells via targeting pathways that inhibit apoptotic death of terminally damaged cells has been described. The mechanisms, specificity, and application of senolytics have recently been extensively reviewed (Niedernhofer & Robbins, 2018; Palmer & Kirkland, 2016); therefore, this article is focused mostly on the effects of senescent cell clearance.

Genetic clearance of senescent cells has been achieved by harnessing a genetic kill switch, integrated into the promoter of one of the most prominent markers of senescence—protein p16. Three genetic variants of this model have become available: p16‐ATTAC (Baker et al., 2011), p16‐3MR (Demaria et al., 2014), and p16‐NTR (Childs et al., 2016). A senescent cell that expresses p16 in the transgenic mouse model also co‐expresses a kill switch protein: FKBP–caspase 9, truncated herpes simplex virus 1 (HSV‐1), thymidine kinase, or nitroreductase, respectively. The kill switches can be activated by small molecules: AP20187, ganciclovir, and metronidazole, respectively. The possibility of senescent cell clearance in the context of damage accumulation raises the following questions.

### 6.1 How does removal of senescent cells affect the level of damage?

There has been no study published so far determining whether senescent cell clearance affects whole‐body damage. Currently available data on the relationship between damage in senescence and aging only allow for building hypotheses to be tested in the future.

The main theme of this article is that senescent cells show an extraordinarily high level of damage, which they acquire during induction of senescence (initial insult‐inducing damage) and after senescence establishment due to an accelerated rate of damage accumulation (Figure 4). Following this hypothesis, elimination of senescent cells should reduce the number of cells with the highest amount of damage. Moreover, elimination of the whole cell would remove types of damage (such as telomere‐associated double‐strand breaks or lipofuscin) that are considered irreparable or nondegradable. Accordingly, reduced frequency of aorta epithelial cells (Roos et al., 2016) and hepatocytes (Ogrodnik et al., 2017) containing multiple TAFs has been detected in old mice upon clearance of senescent cells. Similarly, in mice where senescence has been induced by chemotherapeutics (Demaria et al., 2016) or whole‐body irradiation (Chang et al., 2016), reduction of cells bearing persistent DNA damage was observed upon genetic or pharmacologic clearance of senescent cells. While this line of evidence indicates that elimination of cells bearing the highest amount of damage (i.e., senescent cells) might be possible, it is still uncertain whether it will significantly affect the whole‐body damage level. Abundance of senescent cells in tissues is quite low, estimated at about 5% (TAF‐ and SADS‐positive) in middle‐age mouse liver (Ogrodnik et al., 2017), 20% (SABG‐positive) in old mouse fat (Xu et al., 2015), and 15% (TAF‐positive) in small airway epithelial cells of middle‐age mouse lungs (Birch et al., 2015). Moreover, quantitative measurements of senescent cell abundance can vary depending on the detection method; for instance, frequency of SABG‐positive cells can reach 1.5% (Baker et al., 2016) or 20% (Xu et al., 2015) in 18‐month‐old WT mice as detected by transmission electron microscopy or colorimetric methods, respectively. With such low frequency, elimination of even all senescent cells seems unlikely to lead to the reduction of the cumulative damage levels detected by standard techniques (e.g., Western blot). However, elimination of senescent cells attenuates tissue inflammation (Baker et al., 2016; Schafer et al., 2017; Xu et al., 2015). Reduction in inflammation could originate from the reduction of the secretory profile of the senescent cells, leading to suppression of the bystander effect (Nelson et al., 2012) and, therefore, to the slowdown of damage accumulation. Accordingly, reduction in the amount of DNA damage in nonsenescent hepatocytes and epithelial cells has been detected in liver and media of aorta of old, but not of hypercholesterolemic or obese mice after senescent cell clearance (Ogrodnik et al., 2017; Roos et al., 2016). The aforementioned evidence concerns only DNA damage, as no data yet provide insight into protein or lipid damage upon senescent cell clearance. The main question, that is whether elimination of senescent cells affects whole‐body/organ level of damage, remains to be answered.

### 6.2 Can elimination of senescent cells cure age‐related diseases?

The senolytic approach is aimed at selective elimination of senescent cells, is currently being investigated as a strategy to alleviate various age‐related diseases. A seminal study demonstrated clearance of approximately 30% of senescent cells and improvement of heart, kidney, and adipose tissue function (Baker et al., 2016). Subsequent studies focused on specific age‐related conditions, such as frailty (Baar et al., 2017; Baker et al., 2016; Zhu et al., 2015), idiopathic pulmonary fibrosis (IPF) (Schafer et al., 2017), atherosclerosis (Childs et al., 2016), osteoporosis (Farr et al., 2017), liver steatosis (Ogrodnik et al., 2017), and osteoarthritis (Jeon et al., 2017), where senescent cell clearance proved beneficial, revealing the common denominator of these various, age‐related conditions.

Schafer et al. (2017) showed that SASP may be the key fibrogenic factor in the course of IPF development and that elimination of senescent cells improved pulmonary function, body composition, and physical performance. Another study focused on atherosclerosis (Childs et al., 2016), demonstrating that accumulation of foamy macrophages with senescence markers in the subendothelial space marks the onset of atherosclerosis. Senescent cells were found responsible for the expression of key atherogenic and proinflammatory cytokines and chemokines, facilitating the formation and maturation of atheroma and further through expression of matrix metalloproteases, leading to the rupture of atherosclerotic plaque. Elimination of p16‐positive cells was proven promising as a potential therapy against atherosclerosis (Childs et al., 2016). Bone is yet another organ where cellular senescence was found causal for age‐related pathologies (Farr et al., 2017). In this study, senescent cells were found to impair osteoblast progenitor cell function and bone formation and to increase osteoclastogenesis. In old mice, the senolytic intervention improved bone mass, strength, and microarchitecture, thereby reducing age‐related osteoporosis (Farr et al., 2017). In another study, ectopic accumulation of lipids has been attributed to the senescent phenotype (Ogrodnik et al., 2017). Hepatocytes displaying markers of cell senescence were found to accumulate in aging liver. Impaired mitochondrial function in senescent hepatocytes resulted in the inability to degrade fatty acids and subsequently contributed to liver steatosis. Both pharmacogenetic clearance and pharmacologic clearance of senescent cells were proved to effectively reduce hepatic steatosis (Ogrodnik et al., 2017). Finally, in a recent study, Jeon et al. (2017) have shown the effect of senescent cell removal on the pathology of osteoarthritis. These researchers showed that osteoarthritis in mice leads to the accumulation of proinflammatory, p16‐, and p21‐positive cells at the site of injury. Pharmacogenetic and pharmacologic treatment was shown to decrease articular cartilage erosion and reduce inflammation and the pain associated with osteoarthritis in old mice.

These reports prove that many age‐related pathologies and frailty can be successfully addressed via senolytic therapies. Moreover, introduction of pharmacologic approaches (senolytic drugs) apart from the pharmacogenetic ones requiring specific genetic background (e.g., INK‐ATTAC or 3MR) of the treated animals brings the senolytic strategy closer to the clinic.

### 6.3 Is it possible to slow down the aging process through the clearance of senescent cells?

Aging and age‐related diseases are closely related, and so, the drugs that prolong lifespan can also postpone age‐related diseases (Laplante & Sabatini, 2012; Novelle, Ali, Dieguez, Bernier & de Cabo, 2016). In contrast, disease‐specific drugs do not always extend lifespan. Likewise, elimination of senescent cells that has been shown to alleviate age‐related diseases would not necessarily succeed at slowing down aging. Accumulation of diverse molecular damage in aging cells and extracellular compartments causes a functional failure of tissues, ultimately resulting in death. The process of damage accumulation concerns all cells while only a small proportion of damaged cells become senescent in the process of disease‐free aging. Apart from low senescent cell abundance in the tissue, there might be yet other arguments against the notion that elimination of senescent cells slows down the aging process.

Senescence is usually considered a negative phenomenon, but in the context of disease‐free aging, an alternative might even be more detrimental. A senescent cell can retain at least part of its presenescence phenotype and functionality. An empty space appearing after elimination of a senescent cell is filled out by new cells. This can be achieved by proliferation of either stem or other resident cells that can lead to the depletion of its regenerative potential and replicative senescence, respectively. Consistently, depletion of senescent cells with senolytic agents leads to activation and increased proliferation of hair follicle stem cells (Yosef et al., 2016). Moreover, as senescent cells are involved in the processes such as wound healing (Demaria et al., 2014) and fibrotic scar formation (Krizhanovsky et al., 2008), elimination of those could result in detrimental side effects for the aging organism. Finally, the use of drugs and approaches aimed at clearance of p16‐positive cells imposes a risk of concurrent elimination of cells that have a naturally high expression of p16, possibly independent from cellular senescence. High expression of p16 in nonsenescent cells has been observed for beta cells of pancreatic islets (Helman et al., 2016) and certain subpopulations of neurons (Schmetsdorf, Gartner & Arendt, 2005, 2007). Cellular senescence appears to be a trade‐off between tissue functionality and damage accumulation‐related risk. To address this issue, intermittent, short‐term treatment strategy has been proposed (Niedernhofer & Robbins, 2018; Palmer & Kirkland, 2016).

Up to this day, only a single study showed an increase in mouse lifespan by senescent cell clearance (Baker et al., 2016). The authors reported that C57Bl/6 p16‐ATTAC mice treated with AP20187 from 12 months of age show ~20% increase in lifespan. The data presented, however, indicate that it is the average lifespan that is increased with only a small change in maximum lifespan only under certain conditions (AP20187‐treated males and females on the mixed genetic background combined together). Clearance of senescent cells in this model decreased the risk of death at mid‐ and late life, but overall, did not affect the slope of the mortality curve/rate of aging. The increase in average lifespan is consistent with the decreased prevalence of age‐related diseases in these mice. Similarly, alleviation of the aging phenotype but not the increase in maximum lifespan has been observed in the progeria mouse model BubR1 crossed with p16‐ATTAC and treated with AP20187 (Baker et al., 2011). Interestingly, a recently published study (Xu et al., 2018) showed that senolytic drugs dasatinib and quercetin administered to old mice increase median post‐treatment lifespan (i.e., remaining lifespan) without a significant effect on maximum lifespan reported. Altogether, evidence collected so far does not support the hypothesis that elimination of senescent cells affects the rate of aging/maximum lifespan. It remains to be determined whether this effect results from the lack of impact of senescent cell clearance on the whole‐body damage accumulation.

**Figure 5:**
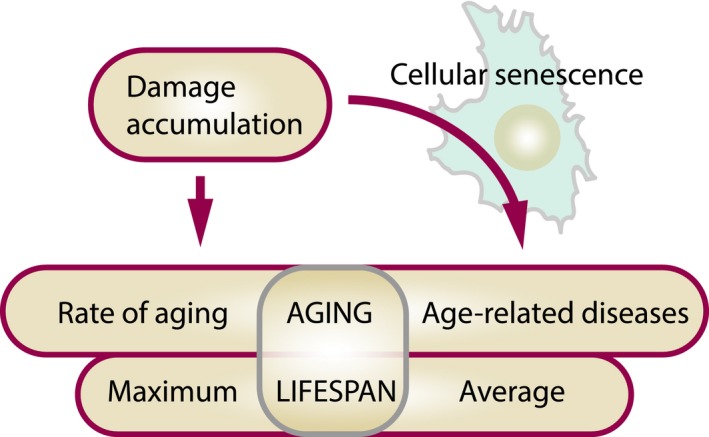
**Damage accumulation accounts for both organismal and cellular aging.** Accumulation of damage causes cellular senescence, which contributes to age‐related diseases and, by doing so, affects the average lifespan. Accumulation of damage can affect the maximum lifespan/rate of aging in a senescence‐independent manner (as damage accumulates in all the cells, not only in senescent) and average lifespan/age‐related diseases through the “damage amplification loop” represented by cellular senescence

Cellular senescence is one of the consequences of the damage accumulation process, but it is not a passively accumulating side effect of aging. On the contrary, the impact of cell senescence can amplify the effect of damage accumulation and go beyond what the wear‐and‐tear effect could do. As damage accumulation primarily defines the aging process, it might not be the direct cause of age‐related diseases. Instead, originating in damage accumulation, self‐amplifying phenomena like cell senescence could be the major driver of diseases in the elderly. In other words, the rate of damage accumulation determines the rate of healthy/natural aging process, while resulting from damage accumulation, cellular senescence can be responsible for the pathologies of aging (Figure. 5).

## 7 CONCLUSIONS

With the recently published evidence, the role of cellular senescence in organismal aging has become increasingly clear. In this review, we integrate the basic mechanistic ideas in the aging field with the novel discoveries in the field of cellular senescence. We put new insights resulting from this work into the perspective of molecular damage and propose models that may be tested in future studies:

1. The phenomenon of cellular senescence has a special meaning in the context of damage accumulation in aging. Cells triggered to senesce by damaging insults exhibit higher basal levels of damaged macromolecules than healthy cells and also generate damage at a higher rate. This notion posits senescent cells as organismal carriers of damage. It is especially relevant for the irreparable forms of damage such as telomere‐associated breaks and lipid–protein aggregates of lipofuscin.
2. Kinetics of senescent cell accumulation in response to lifespan‐modulating interventions differs from the kinetics of irreparable and reparable types of damage. This is due to yet another layer of complexity in the regulation of senescent cell population in vivo that is mediated by the immune system. Subjected to a life‐extending intervention, an organism can remove senescence‐related damage, in contrast to other types of irreparable damage. A change from life‐extending to life‐shortening conditions does not, however, abolish the beneficial effects of the former. As shown for dietary restriction, animals on short‐term DR maintain the status of low senescent cell abundance after the end of the treatment.
3. Accumulation of senescent cells is an integral part of the damage accumulation process. Senescent cells then emerge as causal to age‐related diseases. This model explains the recently published evidence that elimination of senescent cells can alleviate multiple age‐related diseases and increase health span but does not greatly affect the rate of aging/maximum lifespan. As senescent cells contain high levels of irreparable damage, we do not imply that a certain effect on the rate of aging is impossible. However, we argue that elimination of senescent cells is unlikely to be the intervention that would very significantly prolong human maximum lifespan.

We are getting closer to the time when clearance of senescent cells becomes a realistic possibility for treatment of age‐related diseases. At the same time, more and more drugs with senolytic‐like properties are being discovered. However, the road ahead is not as simple, and to comprehensively approach the relationship between cellular and organismal aging for designing therapies as the ultimate goal, cellular senescence should be put into the context of what we know about the aging process and in particular what we know about damage accumulation and age‐related deleterious changes in general. Finally, there are still many gaps and missing links between molecular, cellular, and physiological aspects of senescent cell removal that require experimental studies. This article proposes new experimental approaches to study cellular senescence in the context of organismal aging.

## CONFLICT OF INTEREST

None declared.

## AUTHOR CONTRIBUTIONS

MO wrote the first draft. MO, HS, and VNG extended, revised, and finalized the manuscript.
