# Corrigendum

**DOI:** 10.1111/acel.12942

**Published:** 2019-03-12

Ogrodnik M, Salmonowicz H, Gladyshev VN. Integrating cellular senescence with the concept of damage accumulation in aging: Relevance for clearance of senescent cells. *Aging Cell*, 2019;18:e12841. https://doi.org/10.1111/acel.12841

In the article “Integrating cellular senescence with the concept of damage accumulation in aging: Relevance for clearance of senescent cells,” the corresponding author would like to change his email address from “(m.ogrodnik@newcastle.ac.uk)” to “(Ogrodnik.Mikolaj@mayo.edu).”

The author would like to apologize for the inconvenience caused.

